# Echocardiography and lung ultrasonography for the assessment and management of acute heart failure

**DOI:** 10.1038/nrcardio.2017.56

**Published:** 2017-04-27

**Authors:** Susanna Price, Elke Platz, Louise Cullen, Guido Tavazzi, Michael Christ, Martin R. Cowie, Alan S. Maisel, Josep Masip, Oscar Miro, John J. McMurray, W. Frank Peacock, F. Javier Martin-Sanchez, Salvatore Di Somma, Hector Bueno, Uwe Zeymer, Christian Mueller

**Affiliations:** 1 Royal Brompton & Harefield NHS Foundation Trust, Royal Brompton Hospital, Sydney Street, London SW3 6NP, UK; 2 Department of Emergency Medicine, Brigham and Women’s Hospital, Harvard Medical School, 75 Francis Street, Boston, Massachusetts 02115, USA; 3 Department of Emergency Medicine, Royal Brisbane and Women’s Hospital, Butterfield St & Bowen Bridge Road, Herston, Queensland 4029, Australia; 4 University of Pavia Intensive Care Unit 1st Department, Fondazione Policlinico IRCCS San Matteo, Viale Camillo Golgi 19, 27100 Pavia, Italy; 5 Department of Emergency and Critical Care Medicine, Klinikum Nürnberg, Prof.-Ernst-Nathan-Straße 1, 90419 Nürnberg, Germany; 6 Department of Cardiology, Imperial College London, Royal Brompton Hospital, Sydney Street, London SW3 6NP, UK; 7 Coronary Care Unit and Heart Failure Program, Veterans Affairs San Diego Healthcare System, 3350 La Jolla Village Drive, San Diego, California 92161, USA; 8 Critical Care Department, Consorci Sanitari Integral, Hospital Sant Joan Despí Moisès Broggi and Hospital General de l’Hospitalet, University of Barcelona, Grand Via de las Corts Catalanes 585, 08007 Barcelona, Spain; 9 Emergency Department, Hospital Clínic de Barcelona, Carrer de Villarroel 170, 08036 Barcelona, Spain; 10 BHF Cardiovascular Research Centre, University of Glasgow, 126 University Place, Glasgow G12 8TA, UK; 11 Emergency Medicine, Baylor College of Medicine, Scurlock Tower, 1 Baylor Plaza, Houston, Texas 77030, USA; 12 Emergency Department, Hospital Clinico San Carlos, Instituto de Investigacion Sanitaria del Hospital Clinico San Carlos, Calle del Prof Martín Lagos, 28040 Madrid, Spain; 13 Emergency Department, Sant’Andrea Hospital, Faculty of Medicine and Psychology, LaSapienza University of Rome, Piazzale Aldo Moro 5, 00185 Rome, Italy; 14 Centro Nacional de Investigaciones Cardiovasculares and Department of Cardiology, Hospital 12 de Octubre, Avenida de Córdoba, 28041 Madrid, Spain; 15 Klinikum Ludwigshafen, Institut für Herzinfarktforschung Ludwigshafen, Bremserstraße 79, 67063 Ludwigshafen am Rhein, Germany; 16 Department of Cardiology and Cardiovascular Research Institute Basel, University Hospital Basel, Petersgraben 4, CH-4031 Basel, Switzerland

## Abstract

Echocardiography is increasingly recommended for the diagnosis and assessment of patients with severe cardiac disease, including acute heart failure. Although previously considered to be within the realm of cardiologists, the development of ultrasonography technology has led to the adoption of echocardiography by acute care clinicians across a range of specialties. Data from echocardiography and lung ultrasonography can be used to improve diagnostic accuracy, guide and monitor the response to interventions, and communicate important prognostic information in patients with acute heart failure. However, without the appropriate skills and a good understanding of ultrasonography, its wider application to the most acutely unwell patients can have substantial pitfalls. This Consensus Statement, prepared by the Acute Heart Failure Study Group of the ESC Acute Cardiovascular Care Association, reviews the existing and potential roles of echocardiography and lung ultrasonography in the assessment and management of patients with acute heart failure, highlighting the differences from established practice where relevant.

Heart failure is the primary cause of hospital admission in >1 million patients per year in the USA, with 25% of patients being readmitted within 1 month, and 10–20% mortality at 6 months after discharge^1,2^. Acute heart failure (AHF) — either a new diagnosis in patients with no history of cardiac disease, or as a result of acute decompensation in patients with known heart failure — is the leading cause of hospital admission in individuals aged >65 years in the UK^3^. According to data from Europe, approximately 50% of these patients will be readmitted within 12 months, and 30% will be deceased at the 1-year follow-up^4^. Despite numerous clinical trials to assess optimal treatment and management strategies for patients with AHF, little improvement has been made in AHF outcomes in the past 30 years^1,4,5^, with management decisions largely based on expert consensus rather than robust evidence. The burden of AHF is therefore substantial, both to individual patients and to society^6,7^. The successful management of patients with any acute condition involves early diagnosis, the identification of underlying reversible causes, and the implementation of effective therapies in a timely manner, all while avoiding harm; all these factors are associated with better in-hospital and short-term prognosis^8^. This Consensus Statement, prepared by the Acute Heart Failure Study Group of the ESC Acute Cardiovascular Care Association, reviews the existing and potential roles of echocardiography and lung ultrasonography (LUS) in the assessment and management of patients with AHF.

## AHF: a diagnostic and management challenge

AHF is a syndrome rather than a diagnosis *per se*, caused by a wide array of pathologies that result in a spectrum of disease severity ranging from breathlessness to cardiogenic shock or cardiac arrest. AHF is a highly lethal condition, and studies have shown that minimizing the ‘time to appropriate therapy’ — the initiation of treatment as soon as possible, including in the prehospital setting — is potentially beneficial in improving outcomes^9,10^. AHF is variably defined as the rapid onset or acute worsening of symptoms and signs of heart failure that is associated with elevated plasma levels of natriuretic peptides^4,11^. However, substantial diagnostic uncertainty is inevitable when relying only on traditional clinical findings, and currently a lack of specificity exists in routine investigations for this condition. Indeed, although patients often present with a suggestive history, clinical features (such as shock, and pulmonary or peripheral congestion), and/or symptoms related to the underlying potential cause, these traditional clinical features are frequently absent; over-reliance on these factors might delay diagnosis and implementation of appropriate therapy, or contribute to a missed diagnosis in up to 20% of patients^12,13^. Furthermore, patients’ clinical features might vary according to the site of initial medical contact and the management strategies employed^14,15^.

The majority of patients with AHF present to emergency departments; however, many patient are also assessed and managed in other acute care settings such as in intensive care and inpatient cardiology units. Patients with AHF usually present with symptoms of congestion and breathlessness rather than cardiac arrest or shock^16^. Symptoms of breathlessness account for 3–5% of emergency department attendances in Europe and the USA, and the major causes of breathlessness and their prevalence include AHF (50%), pneumonia or bronchitis (20%), exacerbation of chronic obstructive pulmonary disease or asthma (20%), and pulmonary embolism (5–10%)^16,17^. Current guidelines recommend that clinical examination and investigations should be integrated to form the diagnosis, including the use of electrocardiogram (ECG), chest radiograph, and biomarkers such as natriuretic peptides, troponin, and D-dimer as indicated^16,18,19^. Unfortunately, these data can be challenging to interpret, in particular in the 10–15% of patients in whom two concomitant diagnoses exist^1,4,20^. Specifically, although included in the current definition of AHF, levels of natriuretic peptides can be elevated in respiratory disease and other acute conditions such as pulmonary embolism, sepsis, and anaemia^21–24^.

Any acute condition can be further complicated by the external factors present in emergency settings, such as high ambient noise and restrictive space, limiting a clinician’s ability to position the patient optimally for examination. Furthermore, the frequently atypical features of very severe pathology (in particular valvular disease), and the time pressures imposed by an acutely deteriorating patient can contribute to poor outcomes. These factors are further confounded by the presence of concomitant pathologies in the increasingly ageing patient population^25^.

**Figure 1:**
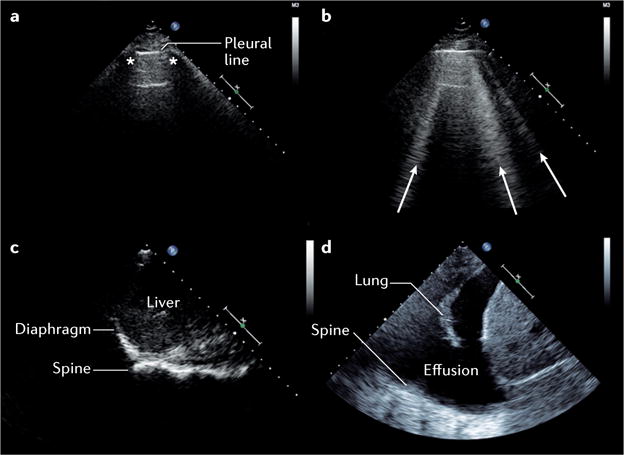
Lung and pleural ultrasonography **a** | Normal lung with pleural line, and ribs (*) with shadowing. **b** | Pulmonary oedema with multiple vertical B-lines (arrows) arising from the pleural line. **c** | Diaphragmatic view with spine ending at the level of the diaphragm, with no pleural effusion. **d** | Pleural effusion seen as anechoic (echo-free) space above the diaphragm with atelectatic lung. Spine can be visualized beyond the diaphragm owing to the effusion.

**Figure 2:**
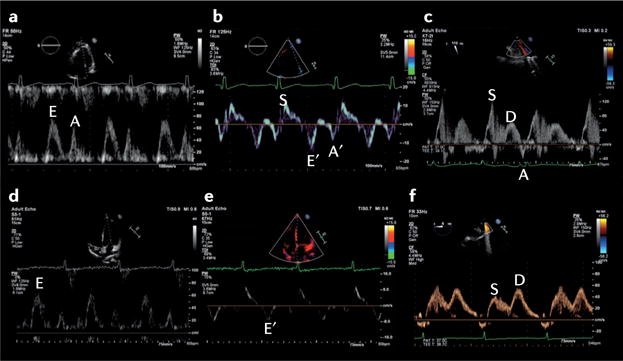
Echocardiographic methods to estimate left atrial pressure The upper panels show the echocardiographic scan of a patient aged 45 years admitted to hospital with dyspnoea owing to severe acute respiratory failure. **a** | Transthoracic echocardiogram (TTE) of the mitral inflow pattern showing a normal early (E) and late (A) transmitral flow pattern. **b** | Tissue Doppler imaging (TDI) of the lateral mitral valve annulus from the same patient; S is systolic annular velocity, E′ is early annular diastolic velocity, and A′ is late annular diastolic velocity (related to atrial contraction). **c** | Pulmonary venous Doppler (transesophageal echocardiography) demonstrating a dominant systolic wave (S) and smaller diastolic wave (D), with a normal deceleration time. The E/A ratio is >1 and the E/E′ is <8 cm/s with a dominant S wave on pulmonary vein, consistent with a normal left atrial pressure. The lower panels show the echocardiographic scan of a female patient aged 59 years admitted with dyspnoea owing to severe left ventricular dysfunction with pulmonary oedema. **d** | TTE of the mitral inflow pattern showing a dominant E wave with E/A ratio >2. **e** | TDI of the septal mitral valve annulus with a very low early diastolic velocity (E′), and **f** | pulmonary venous Doppler (transoesophageal echocardiography) showing a blunted systolic wave (S) and dominant diastolic wave (D). The E/E′ is 16.3 cm/s, and dominant D wave on pulmonary venous Doppler with D deceleration time <150 ms are consistent with an elevated left atrial pressure.

Echocardiography and LUS are readily available and widely validated techniques that can be used to reveal anatomical and physiological abnormalities in patients with AHF, which when correctly applied in the acute setting, can improve patient assessment, management, and outcomes (FIGS 1,2)^26^. Unlike other biomarkers used in AHF, echocardiography and LUS can be used to identify not only inadequate cardiac output and/or the presence of congestion, but also the underlying cause, allowing the most appropriate, individualized interventions to be delivered immediately to the patient^27^. Furthermore, these imaging modalities can be used to monitor the effects of treatment (either beneficial or detrimental), as well as to guide patient disposition and interventions as indicated^28^. Pocket-sized echocardiography devices are practical for screening, and provide information to clinicians in addition to that gathered from auscultation by a stethoscope alone. When AHF is suspected, an integrative approach is recommended, including determination of cardiopulmonary instability and evaluation of congestion (pulmonary and peripheral) using a combination of techniques^4^. When image quality is inadequate, either transoesophageal echocardiography or the use of contrast should be considered.

## Lung ultrasonography

Based on the interpretation of a number of artefacts, specific ultrasonography appearances, and their distribution (FIG. 1), LUS allows for a rapid point-of-care evaluation of a number of conditions, including pulmonary oedema, lung consolidation, pleural effusion, and pneumothorax^29^. High intra-rater and inter-rater reproducibility, ease of learning, short exam duration (<5 min), and the noninvasive nature of this technique makes it an advantageous point-of-care tool^30–32^. LUS is increasingly used in the acute care setting, and has improved diagnostic accuracy compared with clinical assessment and chest radiography for the identification of a cardiac aetiology in patients presenting to the emergency department with undifferentiated dyspnoea^33^.

### Interstitial fluid and pulmonary oedema

Quantification of B-lines (vertical artefacts that result from an increase in interstitial density; FIG. 1b) has been shown to be useful for the diagnosis, monitoring, and risk assessment of patients with known or suspected AHF^34–36^. Either curvilinear or phased array transducers can be used, typically at an imaging depth of 18 cm. Although the assessment of eight or more anterior and lateral thoracic zones (four on each hemithorax) has been recommended in a consensus statement^29^, a subsequent study demonstrated high diagnostic accuracy with examination of only six thoracic regions^33^. The visualization of three or more B-lines in two or more intercostal spaces bilaterally should be considered diagnostic for pulmonary oedema, with sensitivity of 94% (95% CI 81–98%) and specificity of 92% (95% CI 84–96%)^33,37^. By contrast, physical examination and chest radiography have a sensitivity of only 62% (95% CI 61–64%) and 57% (95% CI 55–59%), and a specificity of 68% (95% CI 67–69%) and 89% (95% CI 88–90%) for a diagnosis of pulmonary oedema, respectively^38^. The presence of multiple bilateral B-lines in AHF has been well-correlated with natriuretic peptide levels, and only variably correlated with pulmonary capillary wedge pressure and measures of extravascular lung water^30,33,35,39–41^. Given that studies to assess the incremental diagnostic value of LUS compared with natriuretic peptides for the identification of AHF in patients with dyspnoea reported variable results in different cohorts, this topic warrants further investigation^31,33,42^. The number of B-lines is thought to decrease with treatment for AHF and, therefore, this technique is potentially useful in the monitoring of pulmonary oedema in response to therapy^35,36^. For serial assessments, patient positioning (sitting versus supine) should be kept consistent^43^. Importantly, a higher number of B-lines on LUS at the time of discharge from hospital might help to identify patients with heart failure who have a worse prognosis^36^.

### Pleural effusion

Similarly to B-lines, the presence of pleural effusions can be assessed using curvilinear or phased array transducers in the posterior–axillary line^34^ (FIG. 1d). Current data regarding the diagnostic utility of pleural effusions identified on ultrasonography in patients with AHF are less robust, but have been reported with sensitivities of 79–84% and specificities of 83–98% in small studies of patients with dyspnoea^44,45^.

### Pneumothorax

LUS can be used to exclude pneumothorax in the area scanned with higher sensitivity than supine chest radiography by recognizing lung sliding, a slight horizontal movement of the pleural line with respiration; see Supplementary information S1 (video)^46^. In the setting of a pneumothorax, lung sliding is absent in the affected area of the chest. At the border of a pneumothorax, a transition point between normal lung surface (with lung sliding) and pneumothorax (without lung sliding) can sometimes be identified^47^. This so-called ‘lung point’ confirms the diagnosis. Lung sliding might be absent in several other pathological conditions (such as pleural adhesions or selective mainstem intubation) and, therefore, should not be used in isolation to make the diagnosis of pneumothorax, but rather in conjunction with the full range of sonographic features^46^.

### Differential diagnosis and potential pitfalls

The major questions when using LUS for the assessment of patients with possible AHF include whether there is evidence of pulmonary oedema (such as multiple bilateral B-lines), whether there are other findings suggestive of AHF (such as pleural effusion), and finally, whether there are findings of alternate or concurrent conditions (such as pulmonary consolidation or pneumothorax). Despite its apparent simplicity, a number of caveats exist for the use of LUS. First, B-lines can resolve rapidly in response to treatment, and, therefore, LUS data must be interpreted in the context of previous interventions^35^. Second, B-lines can be seen in a number of pulmonary conditions, including pulmonary fibrosis or interstitial lung disease, acute respiratory distress syndrome, and pneumonitis^29^. The observation of B-lines together with other LUS abnormalities might indicate that two pathologies coexist, or that the B-lines are an expression of pathology other than AHF (for example, acute respiratory distress syndrome, or pulmonary oedema in patients receiving haemodialysis)^48^. Third, large pleural effusions might interfere with B-line quantification in the affected thoracic zones and induce lung consolidation (FIG. 1d). Together, these considerations outline why LUS should not be used in isolation, but rather integrated into clinical and laboratory assessment^33,49,50^.

## Echocardiography in AHF

Driven by progressive advances in ultrasonography technology and an expanding evidence base, the use of echocardiography has extended beyond the traditional application in stable patients to become widespread in the acute and emergency settings^51,52^. Mirroring the concept of critical care, echocardiography is increasingly used as a tool to guide management of the most acutely unwell patients wherever they present along the management pathway. Pocket-sized devices have been recommended in the emergency department, intensive care unit, and coronary units for fast initial qualitative screening of ventricular and valvular function, pericardial and pleural effusion, or extravascular lung water. However, owing to the known limitations of this technique, they are not intended as a substitution for comprehensive echocardiography^26,53^. Remote expert review of images is now a possibility, and in the future, telemedicine will probably have an important role in guiding the assessment and management of these acutely unwell patients.

Echocardiography is used in AHF to help to confirm diagnosis, delineate potential underlying causes, identify associated pathophysiology, and monitor the response to therapy^28,54^. Echocardiography can also be used to guide specialist interventions in the catheter laboratory or operating room^55–57^. Furthermore, echocardiography can address several major questions, including whether a patient has a cardiac cause for their symptoms and signs, the severity of the cardiac impairment and its physiological effect, whether there is an underlying reversible cause, what the most appropriate initial treatment is, and how the patient responds to treatment.

Guidelines recommend immediate echocardiographic assessment for patients with suspected AHF with haemodynamic instability^1,4^; however, interpretation of echocardiographic data in these acutely unwell patients can be extremely complex (TABLE 1). First, the finding of a structurally or functionally abnormal heart does not necessarily mean the cause of dyspnoea is cardiac-related. Second, patients might be misdiagnosed as having primary respiratory disease, even in the presence of very severe cardiac pathology^27,58^. Third, substantial cardiac and respiratory disease might coexist, and determining the degree of cardiac contribution is frequently challenging in this setting^59^. These considerations are further compounded by the relative paucity of high-quality evidence to support the use of echocardiography techniques in the acute arena, as they have been predominantly validated in the outpatient clinic.

### Left-sided disease and elevated LAP

Dyspnoea resulting from left-sided cardiac disease is likely to be associated with elevated left atrial pressure (LAP) and pulmonary oedema. Historically, pulmonary capillary wedge pressure has been measured using a pulmonary artery catheter as a substitute for LAP measurement^60–62^. The use of the pulmonary artery catheter has greatly declined over the past decade, owing to a number of studies that showed potential harm or no improved outcomes in the perioperative and critical care settings^63^. Although absolute pressure values cannot be measured using echocardiography, a drive has occurred to find an echocardiography-derived parameter that can be used to estimate the LAP noninvasively. Indices that have been proposed include interrogation of the transmitral left ventricular (LV) filling pattern (E/A ratio, E wave deceleration time, and the isovolumic relaxation time), pulmonary venous Doppler diastolic deceleration time (FIG. 2), M-mode colour Doppler propagation velocities, the time interval between the onset of early diastolic mitral inflow (E) and annular early diastolic velocity (e′) by tissue Doppler imaging, and the E/e′ ratio^64–69^. None of these measures has been well-validated in the context of emergency medicine^70,71^; they all present technical challenges that must be carefully considered for accurate interpretation, and provide only estimates of a potential range of corresponding LAP values. Even when used in combination (as proposed in critical care), they can at best only indicate that the LAP is probably very high or normal.

LV ejection fraction has been the main parameter used for the diagnosis, treatment, and stratification of patients with heart failure. However, this parameter has several limitations that are particularly relevant in the acute setting, such as load-dependency and inotropydependency^72,73^. Even in the absence of high-quality 2D images, Doppler abnormalities in transmitral filling might provide an early indicator of important pathology^72,74–76^.

Unlike LUS, echocardiography might be challenging to perform well and interpret accurately, as a number of considerations add to the complexity of its application in the acute setting. First, in all parameters described for LAP estimation, the confounding factors imposed by critical illness (changes in heart rate, cardiac output, LV compliance, and volume and ventilatory status) have not been fully evaluated. Second, not only might patients with a relatively normal LAP have radiographic and sonographic evidence of pulmonary oedema, but conversely, patients with chronically elevated LAP might have no evidence of pulmonary oedema. Similarly to LUS, however, the echocardiographic findings should be integrated with those from clinical examination, laboratory investigations, and lung imaging data (radiographic and/or sonographic), and be assessed within the clinical context. The main value of echocardiography in this setting is to diagnose or exclude an underlying cardiac cause for dyspnoea and guide subsequent interventions.

### Right-sided disease: pulmonary embolism

**Figure 3:**
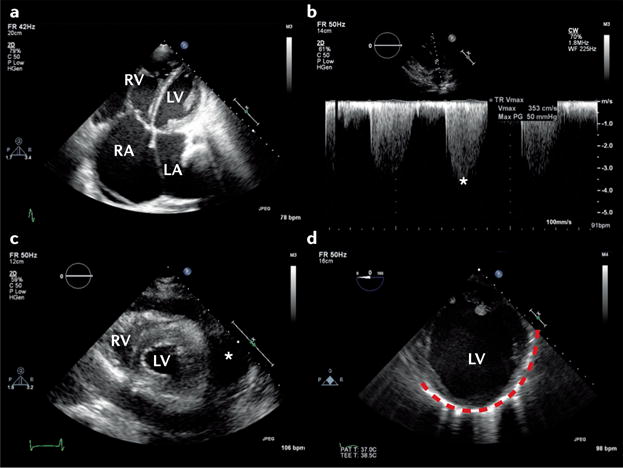
Echocardiographic features in patients presenting with severe haemodynamic impairment **a** | Transthoracic echocardiography in a patient with acute-on-chronic pulmonary embolism from an apical four-chamber view showing a severely dilated right ventricle (RV), and **b** | increased pulmonary systolic pressure estimated by applying the simplified Bernoulli equation using the measured tricuspid regurgitation peak velocity (50 mmHg; asterisk). **c** | Parasternal short axis view showing RV and left ventricle (LV) surrounded by a circumferential pericardial effusion (asterisk) that induced tamponade. **d** | Transoesophageal echocardiography (transgastric short-axis view) of a patient aged 42 years admitted with cardiogenic shock presenting with ST-segment elevation in the anterolateral electrocardiogram leads. Coronary angiography showed critical three-vessel coronary artery disease. The LV is severely dilated, and there is evidence of previous myocardial infarction, shown by the presence of thinned and akinetic myocardium (dotted red line). LA, left atrium; RA, right atrium.

The diagnosis of pulmonary embolism can be challenging, because symptoms and signs are nonspecific. The transthoracic echocardiogram is normal in approximately 50% of unselected patients with acute pulmonary embolism, and has a sensitivity of 50–60% and specificity of 80–90%^77^. Therefore, other investigations are used to confirm the diagnosis, with echocardiography used as a complementary imaging technique^19^. The principal indirect echocardiographic findings are nonspecific, and include right heart dilatation, right ventricular (RV) hypokinesis (with or without apical sparing), abnormal septal motion, and inferior vena cava dilatation^78^ (FIG. 3a). Secondary tricuspid regurgitation might be present, allowing estimation of pulmonary arterial systolic pressure using the simplified Bernoulli equation^79^ (FIG. 3b). Given that the right ventricle can generate a pulmonary artery systolic pressure of only ≤60 mmHg acutely, a higher pressure suggests a more chronic process (either multiple repeated episodes or chronic pulmonary parenchymal disease, with or without pulmonary embolism)^80^. Although the peak tricuspid regurgitation gradient is the most commonly used parameter to assess pulmonary artery systolic pressure in clinical practice, difficulties in the detection of good tricuspid regurgitation envelope might occur. Pulsed Doppler recordings of pulmonary valve flow acceleration time, pre-ejection period, and ejection time at the RV outflow tract can also be used to estimate pulmonary artery pressure and resistance^81,82^.

### Pericardial collection and tamponade

Echocardiography is pivotal for recognition of the haemodynamic consequences of a pericardial collection (FIG. 3c), allowing demonstration of features of tamponade including right atrial and/or RV diastolic collapse, in addition to guiding pericardiocentesis^83^. A number of potential pitfalls exist when interpreting the echocardiographic features of tamponade in the acute setting. These pitfalls include the effects of positive pressure ventilation (reversal of changes in transvalvular flows) and localized collections, in particular after cardiac surgery when substantial haemodynamic compromise might be present, even in the absence of echocardiographic features of tamponade^84^.

### Monitoring of therapy

Echocardiography is not recommended for the monitoring of therapy in patients with AHF in the absence of cardiogenic shock^4,9,11^, given the complexity of LAP estimation using echocardiography, its lack of association with pulmonary congestion and symptoms, and superiority of natriuretic peptide levels in monitoring response to therapy. An emerging area in which echocardiography might be of use is in risk stratification before discharge from hospital. In patients with AHF with dyspnoea, persistent pulmonary congestion before discharge (demonstrated on LUS) has been shown to be an independent predictor of rehospitalization for AHF at 6 months after discharge^36^.

### Cardiogenic shock

Cardiogenic shock is the most severe manifestation of AHF. Although relatively uncommon, the published prevalence (5% of patients with AHF) varies according to the point of initial contact and management (1–2% of patients with AHF in the prehospital or emergency setting versus 29% in intensive care)^4,9,10,16^. Precise definitions of cardiogenic shock can vary; however, the syndrome generally results from inadequate cardiac output for peripheral organ requirements^85,86^. Cardiogenic shock can manifest as hypotension despite adequate filling (with or without vasopressors), altered mentation, cool peripheries, oliguria, hyperlactataemia, metabolic acidaemia, and low mixed venous oxygen saturation^86^. In addition to standard evaluation of critically ill patients in parallel with resuscitation, echocardiography is mandated immediately in patients with cardiogenic shock, because without identification and treatment of the underlying cause, the outcome is usually fatal^9,85^ (FIG. 3d). Additional information that should be obtained from echocardiography includes estimation of stroke volume and cardiac output levels, because these data can provide guidance on how to maximize the cardiac output at the lowest filling pressures (see Supplementary information S2 (table)). These measurements should be taken during the echocardiogram, and should be performed repeatedly to monitor the response to therapeutic interventions and minimize potentially injurious treatment. Every study must be interpreted in the context of the level of inotropic and ventilatory support, as well as metabolic and arterial blood gas status, because these variables might have profound effects on echocardiographic findings.

#### Assessment of volume status

**Figure 4:**
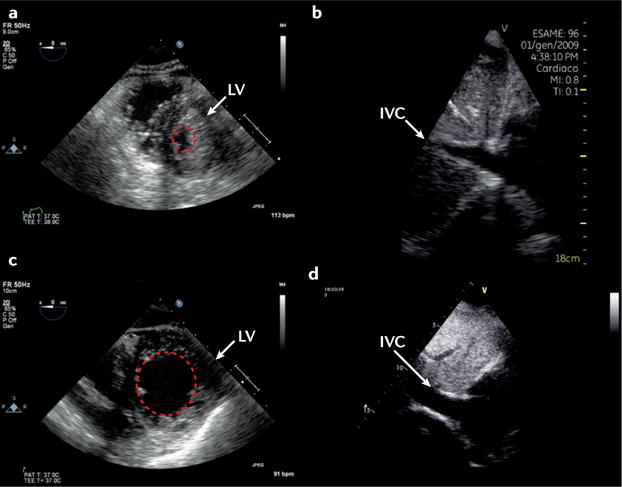
Static 2D echocardiography parameters are used to evaluate potential volume responsiveness The upper panels show a patient who is severely hypovolaemic, and responded to volume loading with an increase in stroke volume. **a** | Short-axis view of the left ventricle (LV) is shown, where the left ventricular end-diastolic area (dotted red circle) is small. **b** | From a subcostal view, an obliterated inferior vena cava (IVC) at end-expiration (<1 cm) can be observed. The lower panels show a patient who, according to static 2D echocardiography parameters, would not be predicted to respond to volume loading by increasing stroke volume. **c** | Short-axis view of the LV with a normal left ventricular end-diastolic area (dotted red circle). **d** | Dilated IVC at end-expiration.

The physiological basis of providing ‘optimal’ filling in cardiogenic shock is that a critical decrease in intravascular-stressed volume reduces the difference between mean systemic venous and right atrial pressure, thereby limiting stroke volume. Although frequently used, invasive static pressure monitoring is not helpful for determining whether an individual patient is volume-responsive^87,88^. Static echocardiographic parameters are widely used to predict volume responsiveness in critically ill patients (FIG. 4); however, their use requires that a number of strict criteria (relating to the patient, their underlying pathology, and medical interventions) are met, otherwise the investigation becomes invalid (see Supplementary information S3 (table)). Similarly, although thought to be superior, dynamic echocardiographic parameters to predict volume responsiveness are valid only in fully mechanically ventilated patients in sinus rhythm and without chronic heart disease^89^. In the presence of cardiac disease (either left-sided and/or right-sided), these measurements can be misleading and should not be used. Conversely, tolerance to volume loading among different patients is variable. The conventional teaching to increase volume in RV failure has not been upheld by findings published in the past 3 years^90,91^. Physiological models suggest that in some patients, progressive fluid loading leads to a plateauing of cardiac output, with a progressive increase in pulmonary artery occlusion pressure. In addition, higher volume is associated with worse outcome in critically ill patients^92–94^.

#### Inotropes and vasoactive agents

Although inotropes and vasopressors are commonly used to improve cardiac output and blood pressure in patients with cardiogenic shock, there is currently insufficient evidence to support the use of any particular agent in this context^9,95,96^. Dobutamine is generally the first-line inotrope of choice in the clinic^9,95,96^. The detrimental effects of positive inotropic agents have been extensively described in the literature^97,98^, and their use should, therefore, be restricted to the shortest possible duration and the lowest dose, both individualized to the patient. Although little guidance exists on how inotrope treatment should be individualized, echocardiography might be helpful in certain scenarios.

**Figure 5:**
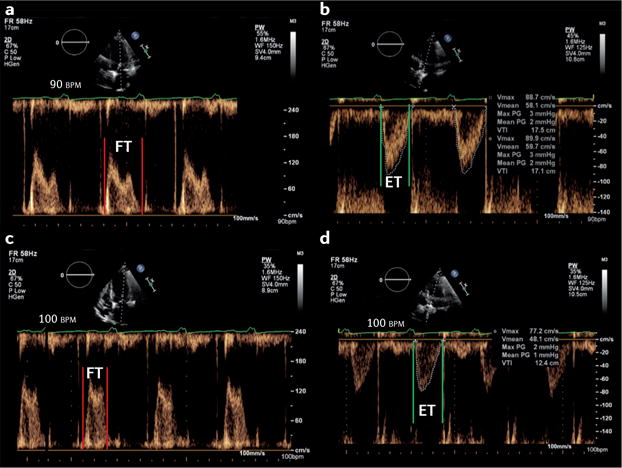
Echocardiography-guided cardiac output optimization using pulsed-wave Doppler imaging **a,b** | Transmitral and transaortic pulsed-wave Doppler imaging at 90 bpm. **c,d** | Transmitral and transaortic pulsed-wave Doppler imaging at 100 bpm. The filling time (FT) is measured from the start to the end of transmitral filling, and the ejection time (ET) from the start to the end of aortic ejection. The total ejection (t–ET) and filling (t–FT) periods are then derived as the product of the corresponding time interval and heart rate, and expressed in s/min. t–IVT (also in s/min) is calculated as 60–(t–FT + t–ET). A heart rate reduction of 10 bpm resulted in a reduction of t–IVT from 16.8 s/min to 10.0 s/min, and a corresponding increase in cardiac output from 3.6 l/min to 5.6 l/min.

**Figure 6:**
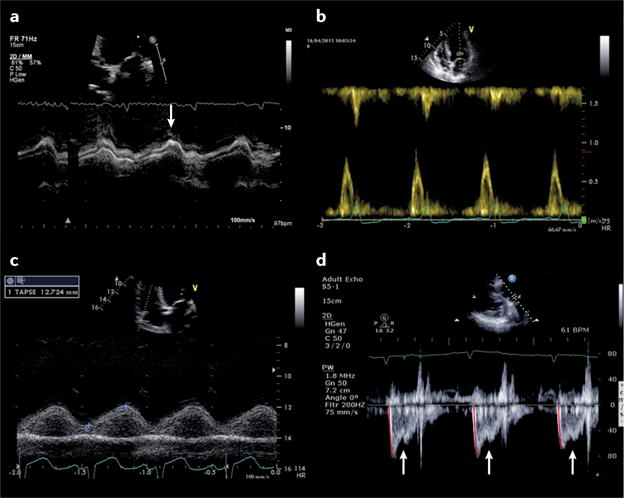
The haemodynamic effects of thrombosis (coronary and pulmonary) as demonstrated by echocardiography **a** | Early features of myocardial ischaemia can be demonstrated by the presence of prolonged long-axis shortening, measured by M-mode echocardiography across the base of the left ventricle (post-ejection shortening; arrow). **b** | Prolonged left ventricular wall tension suppresses early transmitral filling, resulting in an isolated late-diastolic transmitral A wave. **c** | Increased right ventricular afterload leads to a reduction in right ventricular systolic function, as demonstrated by tricuspid annular plane systolic excursion on M-mode echocardiography across the tricuspid annulus. **d** | A substantial increase in pulmonary vascular resistance might be associated with a midsystolic notch (arrows) on pulmonary valve pulsed-wave Doppler ejection wave and a short pulmonary valve acceleration time (78 ms; red lines).

First, not all patients with cardiac disease respond to escalating doses of dobutamine by increasing their stroke volume; in some patients, dobutamine can result in an increase in the total isovolumic time (tIVT)^99^. Echocardiographic identification of an abnormally prolonged tIVT with dobutamine use, or an increase in tIVT in response to escalating inotropic support might indicate that inotropes are directly impairing myocardial performance, thereby prompting a reduction in dose or a change in treatment strategy^99–101^ (FIG. 5). Second, the combination of LV end-diastolic pressure (LVEDP) and low aortic root pressure might result in a mismatch of coronary perfusion and myocardial oxygen demand. If untreated, this mismatch can result in type 2 myocardial infarction^102^ (FIG. 3d). Echocardiographic demonstration of a dominant or isolated A wave on transmitral Doppler in combination with postejection shortening can also be diagnostic (FIG. 6a,b), and indicates that aortic root pressure should be increased and/or LVEDP reduced^103,104^. Third, physiological studies have demonstrated that the combination of RV ischaemia and increased RV afterload is particularly injurious to RV performance, resulting in a fall in systemic blood pressure and cardiac output levels^105^. Echocardiography can be used to estimate pulmonary artery systolic pressure and pulmonary vascular resistance, as well as measure RV dimensions and performance^106^. Echocardiographic identification of high pulmonary vascular resistance with or without pulmonary hypertension in combination with RV dysfunction in cardiogenic shock might necessitate the introduction of a pressor agent plus treatment to reduce RV after-load^90,107^ (FIG. 6c,d). Finally, in a patient with falling cardiac output levels despite escalating inotropic support, echocardiography can help to diagnose LV outflow tract obstruction (with or without associated mitral regurgitation)^27,108^. Treatment in this context involves reduction or cessation of positive inotropic agents, in combination with volume and pressor support.

#### Cardiac arrest

The most extreme presentation of cardiogenic shock is cardiac arrest. International evidence-based guidelines recommend the use of echocardiography to diagnose or exclude some of the causes of arrest^109^. However, echocardiography should not affect the delivery of high-quality cardiopulmonary resuscitation, and specific training in advanced cardiovascular life support is required, even for experienced practitioners. As images are obtained and recorded only during the pulse/rhythm check, studies performed during cardiac arrest are strictly time-limited, and therefore are dissimilar to comprehensive studies that use only focused 2D imaging aimed at diagnosis or exclusion of potentially reversible causes in a simple, binary manner. The pathology leading to arrest is likely to be extreme (tamponade, massive pulmonary embolism, severe LV and/or RV dysfunction, myocardial infarction/ischaemia, hypovolaemia, or tension pneumothorax) and fairly easy to diagnose without more sophisticated echocardiographic techniques. Whether the use of echocardiography in cardiac arrest (and as part of care after resuscitation) can improve outcomes is unknown, but its application in the prehospital setting has been found to change management strategies in up to 60% of patients^110,111^.

#### Acute mechanical circulatory support

**Figure 7:**
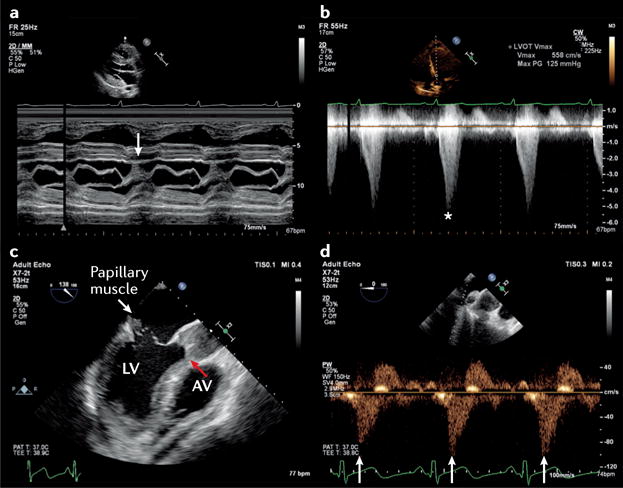
Echocardiographic features in patients receiving extracorporeal support Transthoracic echocardiography in a patient with severe respiratory failure receiving venovenous extracorporeal membrane oxygenation (ECMO). **a** | Parasternal long axis M-mode echocardiography across the mitral valve showing systolic anterior motion of the mitral valve leaflets (arrow). **b** | This motion was associated with substantial left ventricular intracavity gradient of 125 mmHg (asterisk). **c** | A complication of ST-segment elevation myocardial infarction requiring peripheral ECMO is revealed on M-mode echocardiography; papillary muscle rupture had resulted in a flail anterior mitral valve leaflet (white arrow) with associated torrential mitral regurgitation. The increase in left ventricular afterload from ECMO has resulted in failure of the left ventricle (LV) to eject, with a persistently closed aortic valve (AV; red arrow) and stasis of blood in the aortic root. **d** | Reversal of systolic pulmonary venous flow (arrows) in a patient receiving peripheral venovenous ECMO, suggesting inadequate offloading of the LV.

The indications for mechanical circulatory support (MCS) in the acute setting are constantly changing^112,113^. Intra-aortic balloon pumps are no longer routinely recommended for cardiogenic shock^114^. A range of new percutaneous ventricular assist devices are available, in addition to extracorporeal membrane oxygenation (ECMO). These techniques can be used as a bridge to recovery or for longer-term support, and differ not only in terms of their technical aspects, but the degree and type of support provided (LV and/or RV support, with or without the addition of respiratory support)^115–120^. Echocardiography is critical for successful implementation of acute MCS^121,122^ (TABLE 2). MCS is not a treatment *per se*, but instead a supportive therapy for patients awaiting treatment or resolution of the underlying pathological process. As in all cases of AHF, the most important role of echocardiography is to diagnose the underlying cardiac cause. When the decision to institute MCS is made, echocardiography is then used to corroborate the decision regarding the type and level of support required. Although clear echocardiography parameters have been used to guide longer-term MCS for both the left and right heart^123,124^, these parameters are not yet available for devices designed for short-term use. Furthermore, clear contraindications to MCS exist that can be diagnosed only using echocardiography. Echocardiography is used in the initiation of MCS, including the use of vascular ultrasonography to guide safe vessel cannulation and steer device or cannula placement. Echocardiography is subsequently used to monitor MCS by ensuring the goals of support are met, and for detecting complications and assessing tolerance to assistance^121^. Unfortunately, peripheral ECMO can paradoxically worsen cardiac function by increasing LV afterload. Although a number of echocardiographic parameters exist that might indicate this complication (including lack of aortic valve opening, biphasic retrograde flow across the mitral valve in diastole, and retrograde systolic pulmonary venous flow; FIG. 7), the inherent limitations of echocardiography in estimating LAP and LVEDP, especially when the heart is partially bypassed, makes this strategy particularly challenging^122^. Echocardiography can be used, however, to guide interventions to ensure that the heart is adequately offloaded. Finally, a number of echocardiographic parameters are used in conjunction with clinical and haemodynamic assessment to predict which patients might be successfully weaned off MCS^125,126^.

## Other indications

Transoesophageal echocardiograpy can also be used in the acute setting in patients with dynamic mitral regurgitation (see Supplementary information S4 (figure)). Furthermore, features of infective endocarditis caused by aortic prostheses or a device can be demonstrated using transoesophageal echocardiography (see Supplementary information S5 (figure)).

## Quality assurance

A detailed overview of the necessary organizational structure and processes for use of ultrasonography and echocardiography in the acute setting is beyond the scope of this Review, and has been published previously^26,127–130^. However, when used in routine clinical care, training, education, protocols, and ongoing certification of practitioners are required, which should all be performed within existing governance structures.

## Conclusions

Echocardiography and LUS can assist in the rapid assessment of patients with acute dyspnoea and hypotension, and have the potential to transform the way in which clinicians assess and manage critically ill patients with AHF and cardiogenic shock (TABLE 3). The current AHF guidelines are cautious in recommendations for the widespread use of advanced echocardiography techniques in the acute care setting because robust applicability data are lacking, interpretation of findings requires highly specialized, in-depth knowledge of cardiac pathophysiology, and there is potential for harm by injudicious application in this patient population. The opportunities to improve diagnostic accuracy, reduce delays in treatment, and improve outcomes through the use of advanced echocardiography need to be further explored.

## Supplementary Material

Suppl Fig 1

Suppl Fig 2

Suppl Table 1

Suppl Table 2

Video

## Figures and Tables

**Table 1:** Challenges in using echocardiography to determine the underlying cause of AHF

Underlying cause	AHF-related clinical presentation	Echo findings	Notes and potential pitfalls
ACS and ischaemic heart disease	Dyspnoea, as atypical presentation of ACS	Standard RWMA Abnormalities on transmitral Doppler imaging	Transient ischaemia: echo might be normal RWMA not specific for coronary disease Contrast might improve diagnostic accuracy in critically ill patients
	Shock	LV dysfunction	EF influenced by volume, loading, and inotropic status Normal or hyperdynamic left ventricle in unstable AMI implies potential mechanical complication
		Severe MR: primary (papillary muscle rupture and dysfunction) secondary (leaflets normal, but associated with RWMA)	Easy to underestimate degree of LV dysfunction In very severe MR, colour Doppler might underestimate severity Complete or partial papillary muscle rupture Secondary MR can be dynamic
		Ventricular wall rupture: only evidence is pericardial collection (30% of patients)	Detection of pericardial collection should prompt careful scanning for rupture Inferior collection of blood can be challenging to differentiate from liver (similar echo characteristics)
		Ventricular septal rupture: 2D defect in area of infarction with corresponding colour Doppler Can be multiple	Easy to underestimate degree of LV dysfunction and extent of infarction Substantial left-to-right flow in diastole is an indication of high LV diastolic pressure
		RV infarct: features of inferior MI ± RV dyssynergy and paradoxical septal motion	Suspected if TR is low velocity, but PR has steep pressure half-time Assessment of LV function can be challenging, owing to reduced preload Extent of LV dysfunction might be revealed if RV MCS is used
Myocarditis	Widely variable, might be within AHF spectrum	Nonspecific: LV systolic and diastolic dysfunction, resting RWMA, and nonspecific changes in image texture	Additional features: thrombi, secondary MR/TR, pericardial involvement More fulminant: thickening of myocardial walls (oedema) Speckle tracking: reduction in GLS correlates with myocardial inflammation (but nonspecific for the disease) Real-time low-mechanical index MCE might be helpful
Takotsubo syndrome	Widely variable, might be within AHF spectrum	Reversible LV dysfunction with RWMA extending beyond coronary territory distribution	Echocardiographically more heterogeneous than originally described Biventricular involvement in 25% Midsegment involvement in 40%
Dissection	Shock	Dissection flap, varying degrees of AR, and RWMA from coronary involvement	Normal TTE does not exclude dissection AR might be overestimated if dissection flap prolapses through aortic valve
Cardiomyopathy	Full spectrum of AHF	Doppler evidence of elevated filling pressures LUS might show pulmonary oedema	EF influenced by volume, loading, and inotropic status RWMA might occur in absence of coronary disease GLS potentially useful (≤10% indicates severe reduction) GLS and STE not well-validated in acute settings and in the context of positive inotropic agents
		HCM: standard echo features, including estimation of PASP and LAP, plus degree of LVOTO	Severity of LVOTO might be dynamic and worsen with positive inotropic agents and/or hypovolaemia Worsening MR might be dynamic
Pulmonary embolism	Full spectrum of AHF	Dilatation of right heart, RV hypokinesia, abnormal interventricular septal motion Diagnostic: mobile serpentine thrombus in right heart/pulmonary artery	Findings nonspecific for pulmonary embolism Expect to see high PVR In shock, normal right heart virtually excludes pulmonary embolism as the cause Very severe RV dysfunction might underestimate degree of pulmonary obstruction Very severe TR might underestimate degree of pulmonary hypertension
Pneumothorax	From dyspnoea to cardiac arrest	Absence of pleural sliding Demonstration of lung point is diagnostic	If tension pneumothorax suspected in cardiac arrest, treatment should not be delayed for LUS In right mainstem intubation, expect absent lung sliding on left hemithorax
Valve disease	Mitral regurgitation; from dyspnoea to shock	Severity assessed according to standard echo parameters (integrated approach) Underlying causes: ischaemia, endocarditis, trauma, heart failure	Must include cardiorespiratory support: PPV and pharmacological agents can reduce severity significantly Almost always severe in context of papillary muscle rupture Colour Doppler might underestimate severity if valve disease is very severe owing to rapid equalization of pressures Early truncation of MR velocities is a useful sign Suspect in patients with hyperdynamic left ventricle and pulmonary oedema Premature closure of MV (with diastolic MR) implies catastrophic regurgitation If endocarditis suspected, and TTE is nondiagnostic, TOE should be performed
	Aortic regurgitation; from dyspnoea to shock	Severity assessed according to standard echo parameters (integrated approach) Underlying causes: dissection, endocarditis	Short PHT (<200 ms) Diastolic flow reversal in descending aorta (EDV >20 cm/s) Premature diastolic opening of aortic valve implies catastrophic regurgitation Care in evaluation if considering ECMO; even mild degrees of AR might be important (and preclude peripheral ECMO). No aortic valve opening with use of ECMO suggests further LV decompression might be indicated
	Mitral stenosis; might mimic ARDS	Severity assessed according to standard echo parameters (integrated approach)	Acute deterioration might be caused by physiological (pregnancy) or pathological (arrhythmia) precipitant Might see pulmonary infiltrates even in not very severe disease if in combination with lung injury
	Aortic stenosis; from dyspnoea to shock to cardiac arrest	Severity assessed according to standard echo parameters (integrated approach)	Care in evaluation in presence of peripheral ECMO, becausee increase in afterload might reduce aortic valve opening Contraindication to Impella (Abiomed, USA)
				Valve prosthesis dysfunction; from dyspnoea to shock	Echo features of valve dysfunction Underlying causes: thrombus, pannus, endocarditis, dehiscence, degeneration	Normalization of septal motion should raise suspicion Consider if pulmonary infiltrates and ‘good’ or hyperdynamic left ventricle in patient with previous AV/MV replacement Indication for expert TOE Increased transvalvular velocities must be interpreted in context of CO
	Sepsis	Clinically septic, but inadequate CO	Frequently hyperkinetic Pulmonary hypertension: degree of RV dysfunction not uncommon (30%) LV/biventricular dysfunction might occur	If sepsis accompanies pneumonia and venovenous ECMO anticipated, take care to assess right ventricle as it might not tolerate volume load Intracardiac source of sepsis might be present (related to line, device, or valve) Speckle tracking proposed (not validated in adults) to identify early sepsis-related dysfunction
Tamponade	Dyspnoea to shock to cardiac arrest	Demonstration of accumulation of fluid in pericardial space with or without features of tamponade	Small collections occurring rapidly can result in tamponade Localized collections/presence of cardiac or pulmonary disease might suppress features of tamponade Results of postcardiac surgery TTE are frequently negative

ACS, acute coronary syndrome; AHF, acute heart failure; AMI, acute myocardial infarction; AR, aortic regurgitation; ARDS, acute respiratory distress syndrome; AV, aortic valve; CO, cardiac output; Echo, echocardiography; ECMO, extracorporeal membrane oxygenation; EDV, end-diastolic velocity; EF, ejection fraction; GLS, global longitudinal strain; HCM, hypertrophic cardiomyopathy; LAP, left atrial pressure; LUS, lung ultrasonography; LV, left ventricular; LVOTO, left ventricular outflow tract obstruction; MCE, myocardial contrast echocardiography; MCS, mechanical circulatory support; MI, myocardial infarction; MR, mitral regurgitation; MV, mitral valve; PASP, pulmonary artery systolic pressure; PHT, pressure half-time; PPV, positive pressure ventilation; PR, pulmonary regurgitation; PVR, pulmonary vascular resistance; RV, right ventricular; RWMA, regional wall motion abnormality; STE, speckle-tracking echocardiography; TOE, transoesophageal echocardiography; TR, tricuspid regurgitation; TTE, transthoracic echocardiography.

**Table 2:** Echocardiography for acute mechanical circulatory support

Type of mechanical support	Indications	Contraindications	Role of echo
VA ECMO	Cardiogenic shock Inability to wean from cardiopulmonary bypass after cardiac surgery Arrhythmic storm Pulmonary embolism Isolated cardiac trauma Acute anaphylaxis Periprocedural support for high risk percutaneous intervention	Nonrecoverable disease and not suitable for transplantation or VAD Severe neurologic injury or intracerebral bleeding Unrepaired aortic dissection Severe aortic regurgitation	Validation of the underlying cause Biventricular function assessment Guidewire position during cannulation Optimal cannula positioning Postinsertion: Effective LV offloading during ECMO (LV size, LVEDV monitoring if aortic regurgitation is present, aortic valve opening during systole, mitral or aortic regurgitation worsening, biphasic backflow across MV during diastole, retrograde systolic pulmonary flow) Detection of complications (thrombosis, cannula migration, tamponade, intraventricular gradient as per excessive offloading) Weaning from ECMO: assessment of dynamic changes during reduction of ECMO flow (LV and RV systolic function, RV and LV TDI of S′, LV size, LV VTI on aortic valve, mitral and aortic regurgitation, LAP assessment)
Impella (Abiomed, USA)	Additional support for VA ECMO for inadequate offload High-risk PCI and acute MI AMI complicated by cardiogenic shock Acute decompensated ischaemic cardiomyopathy Myocarditis with cardiogenic shock Acute RV dysfunction Bridge to VAD or transplantation Acute ablation of VT (where otherwise nontolerated haemodynamically) Support for BAV (experimental)	Nonrecoverable disease and not suitable for transplantation or VAD Severe neurologic injury or intracerebral bleeding LV thrombus present Ventricular septal defect, or interatrial defect, severe aortic stenosis, and severe aortic regurgitation Mechanical aortic valve Sepsis Bleeding diathesis Severe peripheral vascular disease (left-sided device)	Validation of underlying cause Biventricular function assessment Adequate device position Positioning of inlet and outlet of device Postinsertion: Exclusion of right-to-left atrial shunting Optimization of biventricular filling Detection of complication (cannula thrombus, displacement, inadequate cardiac output, inadequate offloading, failure of the nonsupported ventricle in face of increased forward flow from the supported ventricle)
Tandem Heart (Cardiac Assist, USA)	High-risk PCI and acute MI AMI complicated by cardiogenic shock	Bleeding diathesis Nonrecoverable disease and not suitable for transplantation or VAD Severe peripheral vascular disease	Validation of underlying cause Biventricular function assessment Transeptal puncture Adequate cannula position Postinsertion: Detection of complications (cannula thrombus, displacement, inadequate cardiac output, failure of the nonsupported ventricle in the face of increased forward flow from the supported ventricle)
IABP	Mechanical complication and cardiogenic shock complicating AMI Additional offloading of LV during peripheral VA ECMO Severe MR	Severe peripheral vascular disease Aortic regurgitation	Optimal positioning (TOE, when fluoroscopy not available)

BAV, balloon aortic valvuloplasty; Echo, echocardiography; IABP, intra-aortic balloon pump; LAP, left atrial pressure; LV, left ventricular; LVEDV, left ventricular end-diastolic volume; MI, myocardial infarction; MR, mitral regurgitation; MV, mitral valve; PCI, percutaneous coronary intervention; RV, right ventricular; S′, peak systolic annular velocity; TDI, tissue Doppler imaging; TOE, transoesophageal echocardiography; VAD, ventricular assist device; VT, ventricular tachycardia; VTI, velocity time integral; VA ECMO, venoarterial extracorporeal membrane oxygenation.

**Table 3:** Proposed initial focused cardiac and lung ultrasonography assessment for patients with suspected AHF in acute care setting

Clinical question	Structural and functional assessment	Views (2D imaging)	Comments	Evidence
*Focused echocardiography*^131,132^				
Alternative diagnoses for patient’s signs and symptoms?	Pericardial effusion RV dilatation/systolic function	Subxiphoid, parasternal long-axis and short-axis views, apical four-chamber view	Absence of RV dilatation/dysfunction cannot exclude the presence of pulmonary emboli	Pericardial effusion: sensitivity up to 100%, specificity 95% for detection of pericardial effusion^133,134^ RV dysfunction (various criteria): sensitivity 74%, specificity 54% for diagnosis of acute PE^19^
Evidence of impaired systolic function?	Global LV systolic function	Subxiphoid, parasternal long-axis and short-axis views, apical four-chamber view	Might be useful in new-onset HF for identification of reduced EF	Sensitivity and specificity for diagnosis of AHF depending on prevalence of HFrEF^38,135^
Is there (additional) evidence of volume overload?	IVC assessment	IVC (subxiphoid)	IVC collapsibility <50%	Sensitivity 83%, specificity 81% for diagnosis of AHF in patients with dyspnoea in the ED^135^
Gross structural abnormality as AHF aetiology?	Gross valvular abnormality* Intracardiac mass‡	Subxiphoid, parasternal long-axis and short-axis views, apical four-chamber view	AHF aetiology might be identified in rare cases	NA
*Lung and pleural ultrasonography*^37,38^				
Alternative diagnoses for patient’s signs and symptoms?	Pneumothorax assessment	Anterior, upper chest on each hemithorax	Presence of lung sliding along pleural line rules out pneumothorax in the scanned chest zones	Sensitivity 91%, specificity 98% for detection of pneumothorax^136^
Evidence of pulmonary oedema?	Pulmonary oedema detection	Three or four anterior/lateral chest zones on each hemithorax	Three or more B-lines in two or more zones on each hemithorax considered diagnostic for AHF	Sensitivity 94%, specificity 92% for diagnosis of AHF in patients with dyspnoea in the ED^33,38^
Evidence of pleural effusions?	Pleural effusion detection	Posterior axillary line on both hemithoraces	Echo-free space above the diaphragm	Sensitivity 79–84%, specificity 83–98% for diagnosis of AHF in patients with dyspnoea in the ED^44,45^

* Valvular abnormalities recognizable with focused echocardiography (without the use of Doppler-based techniques) entail leaflet or cusp massive disruption or marked thickening, flail, or anatomical gaps.

‡ Refers to large valve vegetations or visible intracardiac or IVC thrombi. AHF, acute heart failure; Echo, echocardiography; ED, emergency department; EF, ejection fraction; HF, heart failure; HFrEF, heart failure with reduced ejection fraction; IVC, inferior vena cava; LV, left ventricular; NA, not available; PE, pulmonary embolism; RV, right ventricular.

## Key points

- Over-reliance of traditional clinical findings and symptoms can potentially delay diagnosis of acute heart failure (AHF), prolonging the time to appropriate therapy
- The use of echocardiography and lung ultrasonography can help to improve diagnostic accuracy and monitor responses to interventions in patients with AHF
- Lung ultrasonography allows for rapid assessment of numerous conditions, including pulmonary oedema, pleural effusion, and pneumothorax
- Use of echocardiography has extended beyond the traditional application in stable patients to become widespread in the acute and emergency settings
- In the setting of AHF, echocardiography can be used to assess pericardial effusion, right ventricular dilatation, left ventricular systolic function, gross valvular abnormality, and potentially the presence of intracardiac masses
- Echocardiography can also be used to monitor treatment in patients with cardiogenic shock
