# Supplementary material for: Echocardiography and lung ultrasonography for the assessment and management of acute heart failure
Source: Nat Rev Cardiol. Author manuscript; Available in PMC 2018 Jul 1. (PMC5767080; doi:10.1038/nrcardio.2017.56)
Supplement: Suppl Fig 1 [file NIHMS924963-supplement-Suppl_Fig_1.pdf]

In format provided by Price, S. *et al.* (doi:10.1038/nrcardio.2017.56)

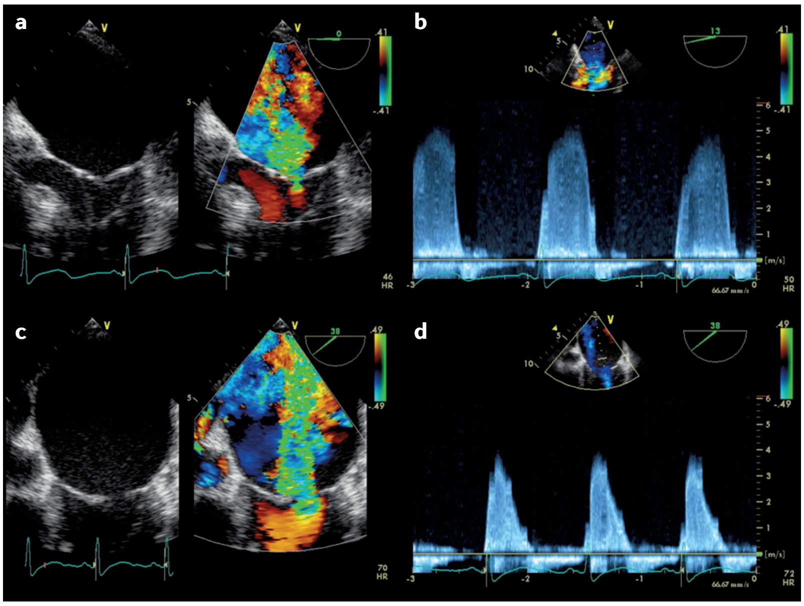

Supplementary figure 1 | **Transoesophageal echocardiography in a patient with dynamic mitral regurgitation.** The patient was hypotensive, with moderate mitral regurgitation shown with **a** | colour Doppler imaging and **b** | continuous-wave Doppler imaging. With volume and pressor loading, the patient developed free mitral regurgitation, with **c** | lack of mitral leaflet coaptation and **d** | a dense, dagger-shaped continuous-wave Doppler signal, with a fall in the pressure difference between the left ventricle and left atrium, confirming the dynamic nature of the regurgitation.

© 2017 Macmillan Publishers Limited, part of Springer Nature. All rights reserved.
