# Supplementary material for: Echocardiography and lung ultrasonography for the assessment and management of acute heart failure
Source: Nat Rev Cardiol. Author manuscript; Available in PMC 2018 Jul 1. (PMC5767080; doi:10.1038/nrcardio.2017.56)
Supplement: Suppl Fig 2 [file NIHMS924963-supplement-Suppl_Fig_2.pdf]

 In format provided by Price, S. *et al.* (doi:10.1038/nrcardio.2017.56)

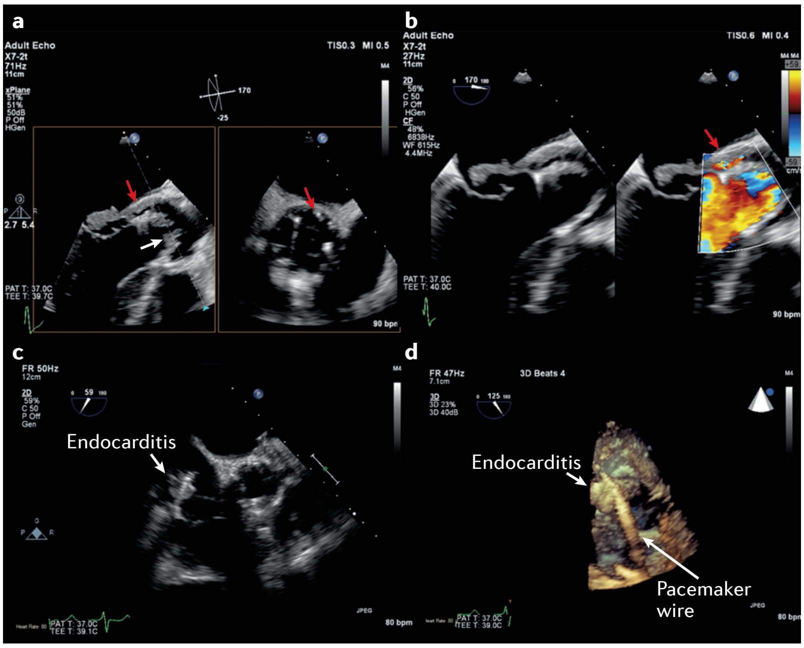

Supplementary figure 2 | **Echocardiographic features of infective endocarditis demonstrated using transoesophageal echocardiography. a,b** | Aortic prosthetic endocarditis showing leaflet thickening (white arrow) and root abscess (red arrows). Lower panels: device-related endocarditis, with vegetations (endocarditis) associated with an atrial pacing wire. Midoesophagel right ventricular inflow–outflow view on **c** | 2D and **d** | 3D imaging.

© 2017 Macmillan Publishers Limited, part of Springer Nature. All rights reserved.
