# Supplementary material for: Echocardiography and lung ultrasonography for the assessment and management of acute heart failure
Source: Nat Rev Cardiol. Author manuscript; Available in PMC 2018 Jul 1. (PMC5767080; doi:10.1038/nrcardio.2017.56)
Supplement: Suppl Table 1 [file NIHMS924963-supplement-Suppl_Table_1.pdf]

In format provided by Price, S. *et al.* (doi:10.1038/nrcardio.2017.56)

Supplementary table 1 | **Common challenges in filling pressure assessment in the acute setting**

Parameter	View and technique	Relevant value(s)	Notes
Isovolumic relaxation time	Transmitral filling (PW Doppler)	• Normal: IVRT 70 + 12 ms<br>• Abnormal diastolic function: IVRT >110 ms<br>• Restrictive physiology: IVRT <60 ms	• Independent of inotropic status<br>• Not widely used (requires phonocardiogram for accurate measurement)
E/A ratio	Transmitral filling (PW Doppler)	• Normal: E/A ratio 0.75–1.40<br>• Abnormal diastolic function: E<A velocity<br>• Restrictive physiology: E>A velocity	• Age-dependent and filling-dependent<br>• Not possible to assess in AF or atrial flutter<br>• Might change in presence of ischaemia/incoordination
E deceleration time	Transmitral filling (PW Doppler)	• Normal: E DT 160–240 ms<br>• Abnormal diastolic function: E DT >240 ms<br>• Restrictive physiology: E DT <160 ms	• Weak negative correlation with PCWP (r = 0.54)<br>• Sensitivity 86%, specificity 59%
E/E′	Transmitral filling (PW Doppler) plus TDI	• Normal: <10<br>• Elevated LAP (septal or average): ≥15<br>• Elevated LAP (lateral): ≥12<br>• PPV: <8 (lateral) predicts PCWP <18 mmHg (sensitivity 83%, specificity 88%)	• If between 10 and 15, will need additional measures<br>• More accurate in patients with reduced EF<br>• Not validated in: MS, MAC, MVR, MR, AF, pacing<br>• Does not necessarily correlate with presence of pulmonary oedema
Pulmonary vein S/D	PW Doppler	• If S/D >0.65 in presence of normal E/A and EF, LAP unlikely to be elevated	• Normal values vary with age
Pulmonary vein DT	PW Doppler	• Calculate LAP<br>• PV–DT <160 ms correlates with PCWP >18 mmHg	• Sensitivity 97%, specificity 96%<br>• Good correlation with PCWP (r = –0.89)<br>• Confounding variables: HR, CO, age, LV compliance, LV afterload, LV relaxation, LAP, PPV
Colour M-mode propagation velocity	Colour M-mode across MV, measure from MV annulus to 4 cm into left ventricle, slope of first aliasing velocity	• <50 cm/s might indicate diastolic disease<br>• PPV: E/Vp <1.7 predicts PCWP <18 mmHg (sensitivity 80%, specificity 100%)	• Difficult to obtain high-quality images in TTE<br>• Fairly low sensitivity
IVC diameter (cm) and collapse with sniff (%)	Subcostal M-mode or 2D echo	0–5mmHg:<br>• ≤2.1 cm, >50% collapse<br>5–10 mmHg:<br>• ≤2.1 cm, >50% collapse<br>• >2.1 cm, >50% collapse<br>+15 mmHg:<br>• >2.1 cm, >50% collapse	• Affected by pressures: intrathoracic, intrapericardial, intra-abdominal<br>• Affected by right heart pathology<br>• Changes in PPV

A, peak late transmitral filling velocity; AF, atrial fibrillation; CO, cardiac output; D, peak pulmonary vein diastolic wave; DT, deceleration time; E, early transmitral filling velocity; E′, peak mitral annular velocity; echo, echocardiography; EF, ejection fraction; HR, heart rate; IVC, inferior vena cava; IVRT, isovolumic relaxation time; LAP, left atrial pressure; LV, left ventricular; MAC, mitral annular calcification; MR, mitral regurgitation; MS, mitral stenosis; MV, mitral valve; MVR, mitral valve replacement; PCWP, pulmonary capillary wedge pressure; PPV, positive pressure ventilation; PW, pulsed-wave; S, peak pulmonary vein systolic wave; TDI, tissue Doppler imaging; TTE, transthoracic echocardiography; Vp, peak aortic velocity.

© 2017 Macmillan Publishers Limited, part of Springer Nature. All rights reserved.
