# Supplementary material for: Echocardiography and lung ultrasonography for the assessment and management of acute heart failure
Source: Nat Rev Cardiol. Author manuscript; Available in PMC 2018 Jul 1. (PMC5767080; doi:10.1038/nrcardio.2017.56)
Supplement: Suppl Table 2 [file NIHMS924963-supplement-Suppl_Table_2.pdf]

Supplementary table 2 | **Common challenges in volume status assessment in the acute setting**

Parameter	View and technique	Relevant value(s)	Notes
Static parameters			
LV end-diastolic area	Short-axis LV on 2D echo	• <5.5 cm^2/m^2	• Might be marker of severe hypovolaemia, but no normal values<br>• Not valid in LV disease<br>• Might appear underfilled as a result of RV dysfunction, not hypovolaemia<br>• Might be affected by intracardiac shunt
IVC end-expiratory dimension	Subcostal view, maximal dimension 1–2 cm from IVC to RA junction on M-mode or 2D echo	• Spontaneous breathing: <1 cm<br>• PPV: <1.5 cm	• Affected by pressures: intrathoracic, intra-abdominal, and intrapericardial<br>• Right heart pathology affects IVC size; might have large IVC, but be profoundly hypovolaemic in the presence of any of these pathologies
Tricuspid TDI	Apical four-chamber on TDI	• Velocity >1.5 cm/s	• Might reflect role of the right ventricle in limiting capacity of the heart to increase SV rather than hypovolaemia *per se*
Dynamic parameters			
ΔPeak aortic VTI	LVOT PW Doppler	>12% indicates up to 15% increase in SV	• Sensitivity 90%, specificity 100%, but false positives likely for high tidal volumes, and false negatives if low tidal volumes
ΔIVC dimensions	Subcostal M-mode or 2D echo	• Spontaneous breathing: %ΔIVC >50% indicates volume responsiveness<br>• PPV (IVC distensibility index) >18% predicts potential 15% increase in SV in response to volume	• Sensitivity 100%, specificity 90%<br>• Unreliable with prominent eustachian valve, large BSA, narrowing of IVC–RA junction, or tissue present in IVC
SVC collapsibility index	TOE 90–100° in mid-high oesophageal view	• %ΔSVC >36% indicates potential 15% increase in SV in response to volume	• Only in patients who are entirely passively ventilated
Passive leg raising ΔSV	LVOT PW Doppler	• SV increase of 12% correlates with fluid responsiveness	• Sensitivity 77%, specificity 100%<br>• Must perform PLR correctly: passive change of patient position from semi-recumbent (45°) to supine<br>• Limitations: profound hypovolaemia, high intra-abdominal pressures, MCS, patient awareness/comfort, RV dysfunction

BSA, body surface area; Echo, echocardiography; IVC, inferior vena cava; LV, left ventricular; LVOT, left ventricular outflow tract; MCS, mechanical circulatory support; PLR, passive leg raising; PPV, positive pressure ventilation; PW, pulsed-wave; RA, right atrial; RV, right ventricular; SV, stroke volume; SVC, superior vena cava; TDI, tissue Doppler imaging; TOE, transoesophageal echocardiography; VTI, velocity time integral.

© 2017 Macmillan Publishers Limited, part of Springer Nature. All rights reserved.
